# Fabrication of Solvent-Free PCL/β-TCP Composite Fiber for 3D Printing: Physiochemical and Biological Investigation

**DOI:** 10.3390/polym15061391

**Published:** 2023-03-10

**Authors:** Sin Ting Ngo, Wei-Fang Lee, Yi-Fan Wu, Eisner Salamanca, Lwin Moe Aung, Yan-Qiao Chao, Ting-Chia Tsao, Hao-Wen Hseuh, Yi-Huan Lee, Ching-Chiung Wang, Wei-Jen Chang

**Affiliations:** 1 Ph.D. Program in Drug Discovery and Development Industry, College of Pharmacy, Taipei Medical University, Taipei 110, Taiwan; 2 School of Dental Technology, College of Oral Medicine, Taipei Medical University, Taipei 110, Taiwan; 3 School of Dentistry, College of Oral Medicine, Taipei Medical University, Taipei 110, Taiwan; 4 Department of Molecular Science and Engineering, National Taipei University of Technology, Taipei 106, Taiwan; 5 School of Pharmacy, College of Pharmacy, Taipei Medical University, Taipei 110, Taiwan; 6 Traditional Herbal Medicine Research Center, Taipei Medical University Hospital, Taipei 110, Taiwan; 7 Dental Department, Taipei Medical University, Shuang Ho Hospital, New Taipei 235, Taiwan

**Keywords:** beta-tricalcium phosphate (β-TCP), guided-bone regeneration, polycaprolactone (PCL), solvent-free, 3D printing

## Abstract

Manufacturing three-dimensional (3D) objects with polymers/bioceramic composite materials has been investigated in recent years. In this study, we manufactured and evaluated solvent-free polycaprolactone (PCL) and beta-tricalcium phosphate (β-TCP) composite fiber as a scaffold material for 3D printing. To investigate the optimal ratio of feedstock material for 3D printing, the physical and biological characteristics of four different ratios of β-TCP compounds mixed with PCL were investigated. PCL/β-TCP ratios of 0 wt.%, 10 wt.%, 20 wt.%, and 30 wt.% were fabricated, with PCL melted at 65 °C and blended with β-TCP with no solvent added during the fabrication process. Electron microscopy revealed an even distribution of β-TCP in the PCL fibers, while Fourier transform infrared spectroscopy demonstrated that the biomaterial compounds remained intact after the heating and manufacturing process. In addition, adding 20% β-TCP into the PCL/β-TCP mixture significantly increased hardness and Young’s Modulus by 10% and 26.5%, respectively, suggesting that PCL-20 has better resistance to deformation under load. Cell viability, alkaline phosphatase (ALPase) activity, osteogenic gene expression, and mineralization were also observed to increase according to the amount of β-TCP added. Cell viability and ALPase activity were 20% higher with PCL-30, while upregulation for osteoblast-related gene expression was better with PCL-20. In conclusion, PCL-20 and PCL-30 fibers fabricated without solvent exhibited excellent mechanical properties, high biocompatibility, and high osteogenic ability, making them promising materials for 3D printing customized bone scaffolds promptly, sustainably, and cost-effectively.

## 1. Introduction

Bone regeneration is always a critical concern for periodontitis patients or after tooth extraction. Several methods and materials have been introduced for bone augmentation or regeneration. In recent decades, clinicians have tended to focus on precision treatment and personalized bone regeneration. Because computed tomography (CT) scans can acquire alveolar bone anatomical data to identify defect areas [1], CT scans and 3D printing techniques can be used to manufacture a customized bone regeneration scaffold carrier that perfectly fits patients’ alveolar bone defect areas. As noted by Yu et al., current knowledge remains limited regarding 3D printing of alveolar ridges, emphasizing the importance of finding osteoinductive and osteoconductive biomaterials that are biodegradable, bioactive, and display desirable mechanical and biological properties that are essential to facilitate bone restoration and regeneration processes [1,2]. In addition, the permeability and porosity of the scaffolds will affect cell migration and vascularization during bone healing. Akbar et al. showed that scaffold permeability can be improved by altering the tortuosity of the scaffold to mimic the structure of cancellous bone [3].

Although guided tissue regeneration (GTR) was introduced in the 1980s, improvements to enhance cell proliferation and osteogenesis while inducing a negligible immune response is needed. Most GTR scaffolds or membranes now used in clinical applications are not custom-made, making a convenient and cost-effective biomaterial for 3D printing a solution to manufacture individualized grafts. Various materials such as metal, polymers, and bioceramics have been investigated for 3D printing [4,5,6], with metallic scaffolds, such as those using titanium, providing biocompatibility and excellent load-bearing support. However, these materials are non-biodegradable, and often necessitate surgery to remove the scaffold after healing. Interest in bioactive biodegradable scaffolds using metals such as magnesium and iron has thus grown. The main drawback of these materials is a rapid degradation rate that has precluded their use in clinical applications [7,8].

Polymer-based scaffolds are another option, being inexpensive, reproducible, and exhibiting great mechanical strength, though most are not osteogenic [9]. Biodegradable polymers are usually non-toxic, induce a low inflammation response, and have relevant mechanical properties and appropriate permeability [10]. Two biodegradable polymers commonly used in 3D printing are polycaprolactone (PCL) and polylactic acid (PLA) [11,12]. Although they are both biocompatible, the by-product of PLA degradation, lactic acid, may induce local inflammation, but this scenario was not reported for PCL.

Bioceramics, with their innate osteoinductivity, have been commonly used for bone regeneration. However, despite their outstanding ability to induce osteogenesis, they show poor mechanical performance and are slow to biodegrade. Bioceramics with calcium compounds such as calcium phosphate and calcium sulfate have shown promise as bone substitutes and are often incorporated into polymers to enhance their mechanical and biological properties [13]. Two calcium phosphate biomaterials commonly used in clinical applications are hydroxyapatite (HAP) and beta-tricalcium phosphate (β-TCP) [14]. HAP, being structurally and chemically similar to bones and teeth, has been well-studied [15]. However, even though HAP is a potent bone regenerator, it is difficult to 3D print. The crucial drawback of using HAP scaffolds in clinical applications is their inability to biodegrade, which can restrict bone regeneration [4]. In contrast, β-TCP is biodegradable and can be replaced during bone healing [16]. The addition of HAP or β-TCP to PLA and PCL composites improved their mechanical properties, making them more suitable for bone tissue engineering [17,18,19].

Fused deposition modeling (FDM) is one of the most used 3D printing methods. To create a scaffold of the desired porosity and size, a printing nozzle is moved along the X, Y, and Z axes [20]. Layer by layer, this FDM printing technique can be used to manufacture a personalized three-dimensional scaffold that precisely fits a defective alveolar bone area [21,22]. A 3D-printed biomaterial scaffold can be easily and reproducibly fabricated with desired physical and chemical properties and used as a carrier that incorporates growth factors, stem cells, or bioceramics while providing sufficient capacity to enhance bone regeneration [23]. Though intriguing investigations have begun into 4D printing techniques whose printed shapes are able to transform over time [24,25,26], 4D printed samples change continuously over time or when exposed to heat, and research into 4D printing currently focuses on prosthodontics and orthodontic applications [26,27].

PCL is a non-toxic, biocompatible, and biodegradable semi-crystalline polyester. Its low melting point of 59~64 °C [28] allows it to blend easily with other polymers and ceramics to alter degradation time and improve surface hydrophilicity and cellular interaction. Research has shown that suitable nozzle temperatures for FDM printing using PCL vary from 100 to 140 °C at pressures of 200–550 kPa [24,29,30,31]. Printing speed, nozzle diameter, and raster angle also influence scaffolds’ mechanical properties [32]. In addition, PCL induces a low immunogenic response and does not accumulate in the human body, making it a suitable biomaterial [33]. However, polymer-based scaffolds are usually bio-inert, hence the incorporation of other bioactive compounds was essential to improve their biological properties. As a bioceramic, β-TCP may be able to enhance osteogenic and angiogenic properties and has been used for dental therapies such as bone regeneration, bone augmentation, sinus lifting, and socket preservation [34,35,36,37,38].

Previous research has demonstrated that PCL/β-TCP promotes bone regeneration better than PCL alone [39]. Baykan et al. illustrated that a PCL/β-TCP composite can induce intramembranous bone formation near defect sites in Wistar rats [40]. Similarly, PCL/β-TCP implanted subcutaneously in rabbit cranial defects showed better angiogenesis and bone reconstruction and did not induce inflammation in defect areas [41]. However, organic solvents such as chloroform or dichloromethane were used to dissolve PCL during the preparation of PCL-based composite [17,42,43]. It has been reported that exposure to chloroform and dichloromethane may irritate the skin, harm the nervous system, and cause cancer.

To the best of our knowledge, no research describes the fabrication of PCL/β-TCP composite fiber without solvent additives. Moreover, the preparation of PCL feedstock used in 3D printing in previous studies required solvent and the material in powder, pellet, or molten forms, which usually requires printing under pressure [29,30,31]. Determining the optimal ratio for fabricating PCL/β-TCP for 3D printing using a solvent-free method for strengthening mechanical properties and enhancing osteoblastic behavior are the important points for bone regeneration. In the present study, a novel blended PCL/β-TCP composite was fabricated using a solvent-free method for sustainability and improving the mechanical properties, biological properties, and clinical convenience in bone regeneration.

## 2. Materials and Methods

### 2.1. Fabrication of PCL/β-TCP Fiber and 3D Scaffold

**Figure 1:**
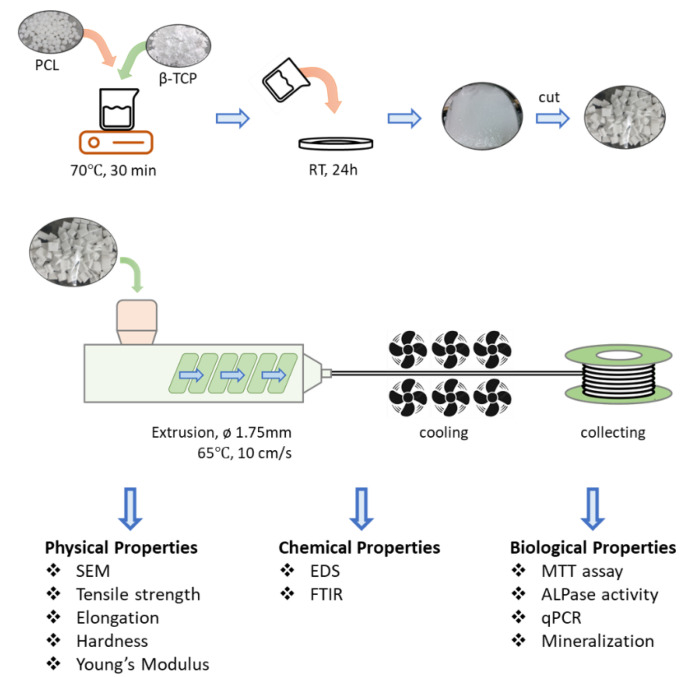
Solvent-free PCL/β-TCP fiber manufacturing process. Different weight ratios of β-TCP were blended into melted PCL at 70 ± 5 °C for 30 min. Evenly mixed PCL/β-TCP was poured onto a plate, spread into a thin layer, and cooled for 24 h at room temperature (RT). The sheet was granulated into small pieces and extruded into 1.75 ± 0.10 mm diameter fibers.

PCL (Mw: 80 kDa; Sigma-Aldrich, Taufkirchen, Germany) and β-TCP (CAS No.: 7758874; Sigma-Aldrich, St. Louis, MO, USA) were mixed at different weight ratios as shown in Table 1. The PCL/β-TCP fiber manufacturing process is illustrated in Figure 1. First, PCL was melted at 70 ± 5 °C for 30 min and blended with β-TCP until the materials were evenly mixed. The PCL/β-TCP mixture was shaped into a thin sheet and cooled for 24 h at room temperature. Later, the PCL/β-TCP sheet was granulated and extruded using a single screw extruder (Filabot EX2 and spooler; Filabot, VT, USA) into fibers with a diameter of 1.75 ± 0.10 mm to ensure that β-TCP was evenly distributed. During extrusion, the processing temperature and extrusion speed were maintained at 70 ± 5 °C and 10 cm/s, respectively. The extruded fibers were immediately cooled with fans to harden the fibers with a consistent 1.75 ± 0.10 mm diameter. The fibers were printed manually into scaffolds using FDM 3D printers (Prusa i3 MK3, Prague) with a printing nozzle temperature of 70 °C and diameter of 1 mm for wettability and mineralization assay.

### 2.2. PCL/β-TCP Material Mechanical Characterization

#### 2.2.1. Scanning Electron Microscopy and Energy Dispersive Spectrometry (EDS) Analysis

The cross-sectional morphology of, and β-TCP distribution in, the PCL/β-TCP fibers were observed with scanning electron microscopy (SEM; SU 3500, Hitachi, Kyoto, Japan). Each specimen was sputter-coated with a 20 nm thick gold layer. Images were taken at 45×, 1500×, and 5000× magnification with an acceleration voltage of 15.0 kV. Secondary electron images (SEI) were taken from at least four random flat areas of the samples at a 1500× magnification, while element composition was determined simultaneously using energy-dispersive X-ray spectrometry (EDS; Quantax EDS, Bruker, Berlin, Germany).

#### 2.2.2. Fourier Transform Infrared Spectroscopy

The functional groups of the PCL/β-TCP composites were analyzed with Fourier transform infrared spectroscopy (FTIR, Nicolet iS5 FTIR Spectrometer, Thermo Fisher Scientific, WI, USA). Each sample was scanned in the transmission mode at a wavenumber of 4000–650 cm^−1^. Peak position and intensity were interpreted to determine the organic compound functional groups in PCL/β-TCP fiber.

#### 2.2.3. PCL/β-TCP Filaments Mechanical Testing

Tensile strength, shore hardness, elongation, and Young’s modulus (MPa) were measured using an AGX-V universal mechanical testing machine (Shimadzu, Kyoto, Japan) in accordance with ISO-37. Each test was performed three times to confirm that mechanical properties could be reproduced. Tensile and elongation specimens were prepared according to ASTM D412, while hardness was evaluated according to ASTM D2240.

#### 2.2.4. Wettability Test

A disk-shaped sample was printed with different PCL/β-TCP fibers. A water droplet was dispensed onto the surface of the sample, then surface wettability was measured using a Goniometer (Digidrop, GBX Scientific LTD, Dublin, Ireland). The analysis software used was Windrop++ (GBX Scientific LTD, Dublin, Ireland). Water droplet images were captured with a camera.

### 2.3. PCL/β-TCP Material Biological Characterization

#### 2.3.1. Preparation of Medium Extraction from PCL/β-TCP Fiber

PCL/β-TCP fibers were extracted with culture medium (Minimum Essential Medium (MEM, Gibco, NY, USA) with 10% fetal bovine serum (Gibco, NY, USA) and 1% penicillin-streptomycin (Gibco, NY, USA)), which was used for biological characterization, where 0.5 g of fiber was exposed to 24 h UV in a 50 mL centrifuge tube prior to extraction. Then 6.5 mL of medium was added for incubation at 37 ± 2 °C for 24 h. The extracted medium was used to determine cell viability, alkaline phosphatase activity, and real-time polymerase chain reaction analyses.

#### 2.3.2. Cell Viability and Biocompatibility

Cell viability was determined using the 3-(4,5-dimethylthiazol-2-yl)-2,5-diphenyltetrazolium bromide (MTT) assay (Invitrogen, OR, USA) by measuring the cellular metabolic activity of MG-63 cell. MG-63 cells were cultured with the extracted medium at a density of 1 × 10^5^ cells per well and incubated at 37 °C (5% CO_2_) for 1, 3, and 5 days. Optical density was measured using a SpectraMax iD3 Multi-Mode Microplate Reader (Molecular Devices, CA, USA) at a wavelength of 570 nm.

#### 2.3.3. Alkaline Phosphatase Activity

MG-63 cells were cultured for 1, 3, and 5 days with the extracted medium at a density of 1 × 10^5^ cells per well, then incubated with Alkaline Phosphatase Activity Colorimetric Assay Kit (BioVision Incorporated, CA, USA), which contains p-nitrophenyl phosphate (pNPP) at 25 °C for 60 min. The pNPP was dephosphorylated into nitrophenol using cellular phosphatase, optical density was observed at 405 nm, and ALP activity (unit/L) was measured.

#### 2.3.4. Real-Time Polymerase Chain Reaction (qPCR)

MG-63 cells were cultured at a density of 1 × 10^5^ cells per well for 1, 3, 5, and 7 days with PCL/β-TCP extracted medium. Then, cellular RNA was collected and transcribed into cDNA using a high-capacity cDNA reverse transcription kit (Applied Biosystems, Vilnius, Lithuania). Fast SYBRTM Green Master Mix (Thermo Fisher Scientific, Vilnius, Lithuania) and LightCycler^®^ 96 System (Roche Inc., Branchburg, NJ, USA) were used to conduct the qPCR test. The PCR-cycling condition was set at 95 °C for 10 min, followed by 45 cycles of 95 °C for 15 s (denaturing) and 60 °C for 60 s (annealing and extension). Finally, gene expression was measured using the delta-delta calculation technique [44]. Osteocalcin (OC), Distal-less homeobox 5 (DLX5), Sp7, osteoprotegerin (OPG), receptor activator of NF-κB (RANK), and alkaline phosphate (ALP) were used as markers, while GAPDH was used as the housekeeping marker.

#### 2.3.5. Mineralization Assay

MG-63 cells were seeded onto the 3D printing scaffold at a density of 1.4 × 10^2^ cells per scaffold for 14 and 28 days. On days 14 and 28, cells were fixed with 70% ethanol before being stained with Alizarin Red S Solution (CAS No.:130-22-3, Sigma-Aldrich, Missouri, USA) for 5–10 min. Samples were washed with deionized water and dried before being photographed. Cells were then destained with 10% Cetylpyridinium Chloride solution (CAS No.: 6004246, Sigma-Aldrich, St. Louis, MO, USA) in a 10 mM Sodium Phosphate Monobasic solution (CAS No.: 7558-80-7, Sigma-Aldrich, St. Louis, MO, USA), shaken for 60 min, and the optical density was observed at 540 nm.

#### 2.3.6. Statistical Analysis

Quantified data are presented as mean ± standard deviation. Averages and standard deviations were calculated using Excel software. Statistical significance was calculated using Student’s t-test, with significance indicated by * for *p*-value < 0.05, ** for *p*-value < 0.01, and *** for *p*-value < 0.001.

## 3. Results

### 3.1. Morphology and Chemical Properties of PCL/β-TCP Fiber

**Figure 2:**
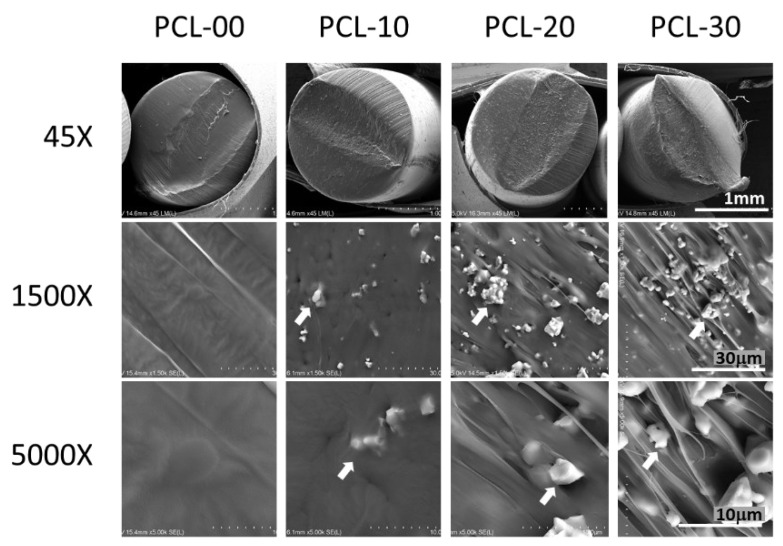
Cross-sectional surface morphology of different PCL/β-TCP ratio fibers using SEM. β-TCP distribution in PCL fibers is indicated by white arrows. The scale bar indicates 1 mm at 45×, 30 μm at 1500×, and 10 μm at 5000×.

**Figure 3:**
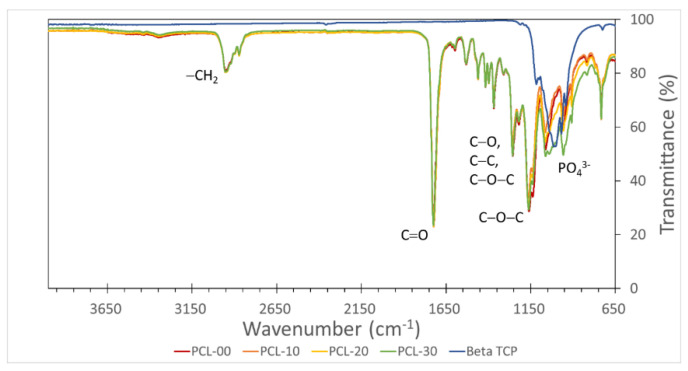
Functional group analysis of PCL/β-TCP using FTIR. PCL, β-TCP, and different ratios of PCL/β-TCP were analyzed to determine their functional groups.

β-TCP particles in PCL/β-TCP fibers were evenly distributed when observed under SEM, as can be seen in Figure 2 where β-TCP particles are indicated using white arrows. The number of β-TCP particles was also observed to increase proportionally to the amount of β-TCP added, demonstrating that the β-TCP particles are uniformly blended into the PCL. Element distribution was further analyzed with EDS. Carbon and oxygen were found in PCL-00 fiber, while calcium (Ca) and phosphorus (P) were detected at increasing levels as the amount of β-TCP increased, with PCL-30 containing the highest percentage of Ca and P (Table 2). The observation under SEM of Ca and P in all groups except PCL-00 verifies that these particles originate from the addition of β-TCP. Functional groups of PCL/β-TCP fibers were analyzed using FTIR (Figure 3). The main prominent peaks for PCL appeared at ~2950–2850 cm^−1^ for asymmetric and symmetric –CH_2_ stretching, ~1720 cm^−1^ for C–O stretching, ~1150–1300 cm^−1^ for C–O and C–C stretching, and asymmetric and symmetric C–O–C. β-TCP peaks appeared at 950~1150 cm^−1^ for vibrational modes of PO_4_^3−^. All PCL/β-TCP fibers displayed peaks that merged with prominent PCL peaks, with a shift occurring at 950~1150 cm-1 when β-TCP particles were added.

### 3.2. Physical and Mechanical Evaluation of PCL/β-TCP Fiber

**Figure 4:**
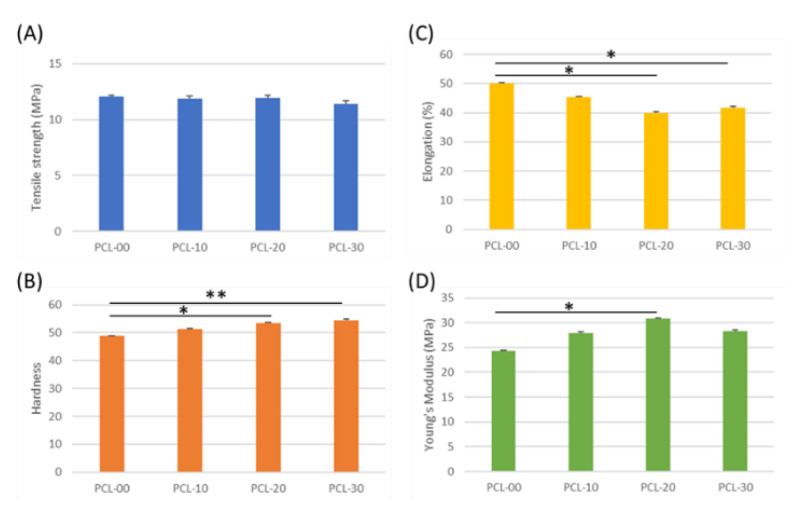
Mechanical analysis of different PCL/β-TCP ratios. (**A**) Tensile strength, (**B**) Shore hardness, (**C**) elongation, and (**D**) Young’s Modulus were measured and calculated. Tensile and elongation specimens were prepared according to ASTM D412, while hard-ness was evaluated according to ASTM D2240. Significance is indicated by * when *p* < 0.05 and **, *p* < 0.01 compared to PCL-00.

**Figure 5:**
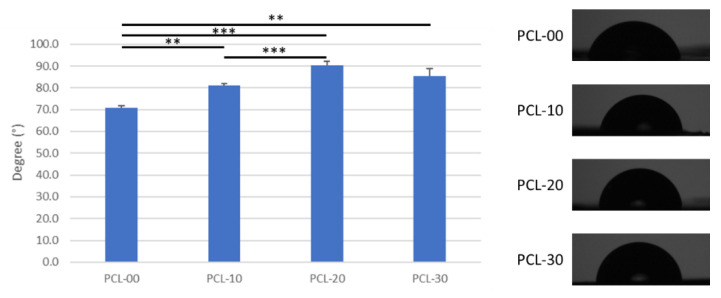
Wettability test of PCL with different ratios of β-TCP. Significant differences are indicated by ** for *p* < 0.01 and *** for *p* < 0.001.

The tensile strength of all PCL/β-TCP fiber groups was similar, with PCL-00 at 12.1 ± 0.2 MPa, PCL-10 and PCL-20 at 11.9 ± 0.2 MPa, and PCL-30 at 11.4 ± 0.3 MPa (Figure 4A). PCL fiber hardness increased according to the amount of β-TCP added, with PCL-30 observed to be 12% harder than PCL-00 (Figure 4B). The addition of β-TCP particles had no impact on tensile strength or PCL fiber hardness. Interestingly, elongation, Young’s Modulus, and wettability patterns did not change in proportion to β-TCP particle content. Elongation for PCL-20 was 10% lower compared to PCL-00 (50.08%) and reverted to 41.8% for PCL-30 (Figure 4C). Figure 4D shows that Young’s Modulus increased significantly from 24.33 ± 2.7 MPa to 30.79 ± 5.6 MPa for PCL-00 and PCL-20, while declining slightly by 4.78 MPa for PCL-30. Low elongation and high Young’s Modulus imply that PCL-20 has better resistance to deformation under load. Moreover, testing showed that PCL-20 had the highest degree of wettability at 90.4 ± 1.8°, while PCL-00 was the most hydrophilic at 70.7 ± 1.2°. The wettability testing results are shown in Figure 5.

### 3.3. Inductive Effect of PCL/β-TCP Composite on MG63

**Figure 6:**
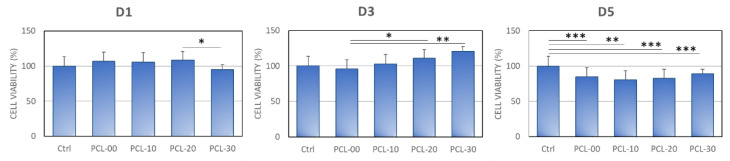
MG63 cell viability when cultured in PCL/β-TCP extraction medium for 1, 3, and 5 days. Significant differences are indicated by *, *p* < 0.05, **, *p* < 0.01 and ***, *p* < 0.001.

Cell viability, differentiation, and mineralization were conducted to study the toxicity and osteogenic properties of the PCL/β-TCP biomaterial. MG63 cell viability was determined with MTT assay (Figure 6). On day 1, there was no significant difference in cell viability across all PCL and control groups, with PCL-30 having slightly lower cell viability than PCL-20. Although PCL-30 showed the lowest cell viability at 95.0 ± 6.8%, this is considered non-toxic according to the ISO-10993 definition of cytotoxicity. By day 3, however, PCL-30 had the highest cell viability followed by PCL-20, with cell survival rates of 120.3 ± 17.6% and 110.9 ± 7.2% respectively. Although cell viability was significantly lower for all PCL groups on day 5 when compared to the control, the overall cell viability of MG63 cells on day 5 was still significantly higher compared to day 1. Even though all PCL groups showed lower cell viability compared to the control group, PCL-30 still presented the highest cell viability among all PCL groups with a cell viability rate of 88.8 ± 4.7%.

**Figure 7:**
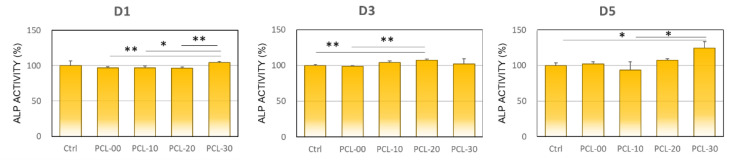
Alkaline phosphatase activity of MG63 cell on PCL/β-TCP extraction medium for 1, 3, and 5 days. Significant differences are indicated by *, *p* < 0.05 and **, *p* < 0.01.

The osteoblastic effect of PCL-30 was slightly higher compared to other groups, with ALPase activity expression of 104.5 ± 1.6%. On day 3, PCL-20 had the highest ALPase activity expression at 107.3 ± 1.5%, followed by PCL-10 (104.0 ± 2.5%) and PCL-30 (102.0 ± 7.5%). The ALPase activity of MG-63 cells peaked at day 5, with PCL-30 at 124.4 ± 9.3% followed by PCL-20 at 107.3 ± 2.6%, as illustrated in Figure 7.

**Figure 8:**
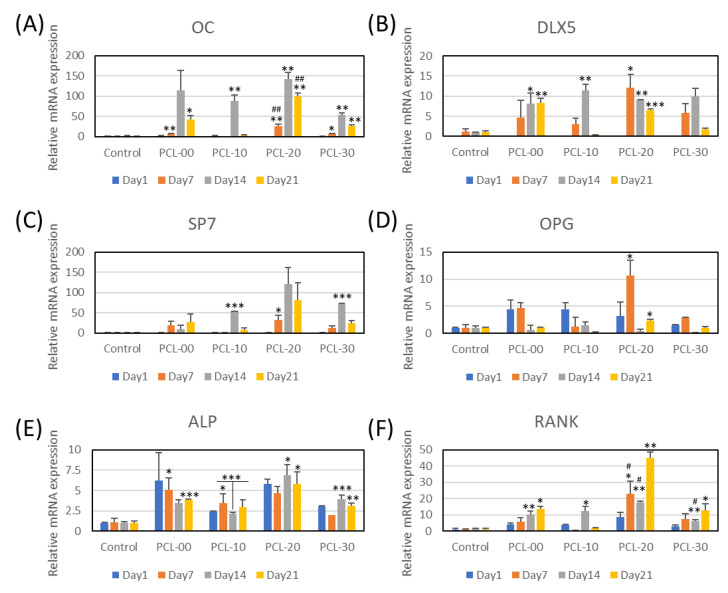
Gene expression of MG-63 cells when cultured in PCL/β-TCP extraction medium for 1, 7, 14, and 21 days. Six different gene markers (**A**) OC, (**B**) DLX5, (**C**) SP7, (**D**) OPG, (**E**) ALP, and (**F**) RANK were analyzed. Significant differences are indicated with *, *p* < 0.05, **, *p* < 0.01, and ***, *p* < 0.001, when compared to control, while the # symbol indicates compared with PCL-00.

Osteogenesis-related gene expression of OC, DLX5, SP7, OPG, ALP, and RANK was analyzed using qPCR for MG63 cultured with PCL fibers for 1, 7, 14, and 21 days (Figure 8A–F). PCL-20 had the highest gene expression for all markers among all PCL/β-TCP fibers. Significantly, mRNA expression of OC, DLX5, and SP7 genes was upregulated on Day 14 and declined on Day 21. The mRNA expression of OPG decreased with time, with the highest expression on Day 1 for PCL-00 and PCL-10, and Day 7 for PCL-20 and PCL-30 (Figure 8D). The gene expression level of ALP fluctuated for all PCL groups. Alternately, ALP and RANK gene expression for all PCL groups was upregulated, with PCL-20 markedly increased on Day 21.

**Figure 9:**
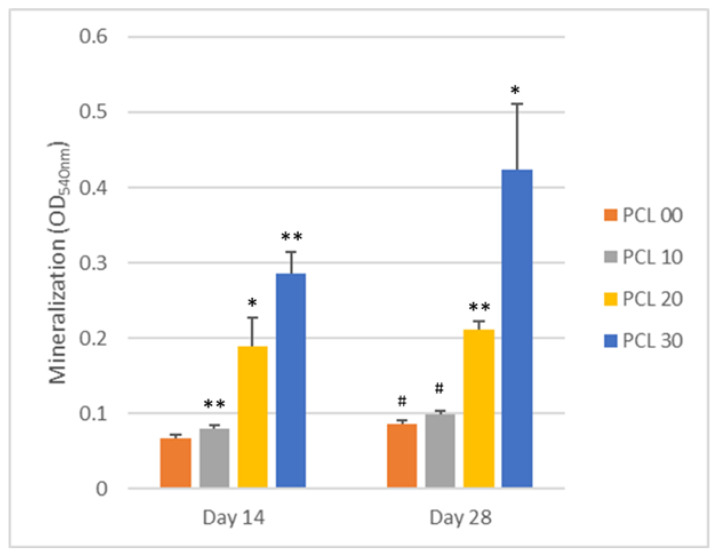
Mineralization assay of MG-63 cells when cultured on PCL/β-TCP scaffold for 14, 21, and 28 days. Significant differences are indicated by *, *p* < 0.05, and **, *p* < 0.01, when compared to the control group on the same day. Significant differences are indicated by #, *p* < 0.05, when compared to the respective group on Day 14.

Because mineralization is an essential process of bone healing, determining the scaffolds’ ability to mineralize is necessary. To do this, MG-63 cells were cultured on PCL or PCL/β-TCP scaffolds for 14 and 28 days (Figure 9). Mineralization ability increased corresponding to the amount of β-TCP added, indicating that PCL/β-TCP composites are a potent bone healing material. PCL-20 and PCL-30 showed a significant increase in mineralization compared to PCL-00 on day 14 and day 28.

## 4. Discussion

PCL has been used as an ingredient in 3D printing raw materials, but with solvents added during the manufacturing process. There has, to date, been limited research into the fabrication of PCL/β-TCP into fibers, instead, PCL must be pre-melted into a liquid state and printed under pressurization. Recent reviews by Francisco et al. and Mohd et al. show that PCL, β-TCP, and HAP are the most reported biomaterials used for 3D printing. However, only a limited number of studies using PCL/β-TCP have been reported, none of which used fabricated solvent-free PCL/β-TCP fibers [15,45]. Wang et al., also note that the fabrication of scaffolds using extrusion methods has been rarely investigated [18]. To our knowledge, no studies have yet reported the use of PCL/β-TCP fibers manufactured using a solvent-free method. In this study, four different weight ratios of PCL/β-TCP fibers were manufactured in an eco-friendly way with no solvent added at any point during the manufacturing process and compared for physical, chemical, and biological characteristics.

PCL has a low melting point, enabling it to be easily formed into different structures at different sizes. Since heating the material could affect its chemical structure, the chemical bonding of the PCL/β-TCP fibers was measured with FTIR to ensure that PCL/β-TCP fibers were chemically stable after heating and extrusion during the manufacturing process. The –CH_2_, C=O, C–O, C–C, and C–O–C chemical bonds were identical for all PCL/β-TCP fibers, suggesting that PCL is chemically intact after heating. PO_4_^3−^ peaks increased with the amount of β-TCP added, indicating that more phosphate particles are found in the PCL/β-TCP fibers. This finding was consistent with SEM imaging of the material.

Tensile strength indicates the maximum force a material can bear before breaking when stretched, while elongation is associated with a material’s deformation. Tensile strength and elongation were found to be inversely proportional to the amount of β-TCP added, a result similar to findings by Bruyas et al. [46]. In addition, Shim et al. found that fabricated PCL and PCL/β-TCP membranes had maximum tensile strengths of 4.9 MPa and 5.1 MPa, respectively [39]. Wang et al. have also shown that the addition of HAP increased compression and elastic modulus, suggesting that PCL/HAP is more resistant to deformation than PCL alone [18]. The tensile strength of the PCL/β-TCP fibers in this study was comparable to the commercial membrane Remaix (10.4 ± 2.7 MPa) and much higher than Bio-Gide (4.6 ± 0.9 MPa), which suggests that our biomaterial may fulfill the mechanical requirements for clinical applications [47]. In addition, Young’s modulus values of our PCL/β-TCP fibers (24.3–30.8 MPa) were within the range of natural mandibular trabecular bone (6.9–199.5 MPa) [48]. Membranes usually need to be secured to surrounding bone during bone regeneration treatment, so high tensile strength with low elongation would prevent the membrane from becoming dislocated from the defect area. β-TCP is generally considered a hydrophilic bioactive material, but research by Inna et al. also illustrates that the water contact angle increases when β-TCP is coated onto the surface of a material [49]. Although a higher amount of added β-TCP increased the water contact angle indicating that the sample surface is more hydrophobic, Morgan and Paul demonstrated that proteins tend to adhere to less wettable surfaces [50]. The slight difference in hydrophobicity had no significant influence on cell attachment or cell viability as observed from the MTT assay. In addition, Shim has suggested that PCL/β-TCP’s hydrophobicity could maintain the scaffold’s structure under wet conditions [39].

Polymer is frequently used together with calcium phosphate to overcome inertness and enhance osteoinductivity. Cell viability increased proportionally with the addition of β-TCP, a finding similar to results by Bruyas et al. who proposed that increased β-TCP could increase surface roughness which facilitates cell growth [46]. Juan et al. demonstrated that MG-63 cells cultured with PCL/β-TCP had better cell proliferation and ALPase activity compared to PCL/HAP and PCL/HAP/β-TCP [51]. Bruyas et al. and Shim et al. have also shown that the addition of β-TCP composite could induce cellular alkaline phosphatase secretion and mineralization [39,46]. Overall, the β-TCP composite is correlated with cell proliferation and differentiation properties.

In order to gain a better understanding of osteogenic differentiation induced by PCL/β-TCP fiber, targeted gene expression of bone markers was determined using q-PCR. To our knowledge, research is limited regarding gene expression induced by PCL/β-TCP biomaterials fiber. Osteocalcin (OC) plays an important role in bone turnover, which is upregulated during bone remodeling [52]. Distal-less homeobox 5 (DLX5) is an essential progenitor, which is associated with Runt-related transcription factor 2 (RUNX2) and alkaline phosphate (ALP) to stimulate bone differentiation [53,54]. Sp7 is known to be involved in the development of chondrocytes and osteoblast maturation. Bone formation still occurred even with Sp7 gene knockout, but delayed bone maturation will be delayed and bone formation unregulated [55]. OC, DLX5, and SP7 produced by osteoblast cells were highly expressed on Day 14, suggesting that new bone formation and bone maturation are occurring. Interestingly, ALP was upregulated throughout the study, especially for PCL-20. Osteoprotegerin (OPG), an activator of NF-κB (RANK) and NF-κB ligand (RANKL) receptors, always work together as the RANKL/RANK/OPG system which regulates bone remodeling by activating osteoclast cells. During bone remodeling, osteoclasts will be activated when RANKL binds onto RANK receptors, and bone reabsorption will occur. However, OPG acts as a negative regulator, which prevents bone reabsorption by binding to RANKL [56]. RANK gene expression is inversely proportional to OPG, where OPG is significantly upregulated on Day 7 while RANK is highly upregulated on Day 21 for PCL-20, indicating the presence of bone remodeling.

Despite these results, there are some limitations during the fabrication of the PCL/β-TCP fibers. In previous studies, PCL composite was fabricated in the presence of solvent; however, under solvent-free conditions, fibers cannot be smoothly extruded if increased amounts of β-TCP are added, thus 30% β-TCP was the maximum weight ratio used in this study. Additionally, most 3D printers on the market have a high-temperature nozzle that runs between 180 °C and 210 °C, but the optimal printing temperature for PCL lies between 60 °C and 140 °C. Accordingly, some modification and troubleshooting will be needed to print PCL scaffolds under optimal printing conditions. To overcome this, some researchers printed scaffolds by pre-heating the PCL composite into a molten state. However, we proposed to fabricate solvent-free PCL/β-TCP fibers that can be printed directly in a more cost-effective and environmentally-friendly manner. The incorporation of β-TCP into PCL significantly improves mechanical properties by increasing hardness and Young’s Modulus. Moreover, bone regeneration is promoted by enhancing the osteogenic properties of osteoblasts. Further characterization of 3D-printed scaffolds will be essential to determine the feasibility of these materials from physiochemical and biological perspectives, including in vivo studies.

## 5. Conclusions

In this research, solvent-free PCL fibers with different β-TCP ratios were manufactured and their physical, chemical, and biological properties were analyzed. All PCL/β-TCP fibers exhibited great mechanical properties, with PCL-20 and PCL-30 being the best performing. Because the fabrication process used in this study was solvent-free, concerns about residual contamination and environmental pollution are reduced. The biological properties of PCL are enhanced with the addition of β-TCP by inducing osteogenic differentiation and gene expression. Finally, the PCL/β-TCP composite in fiber form is more easily used on-site by clinicians or technicians, as the fibers can be prepared in advance. Further research is required to investigate the physiochemical and in vitro and in vivo properties of the 3D-printed scaffolds. In summary, PCL fiber combined with β-TCP at an optimal ratio between 20 and 30% is a potential solvent-free biomaterial for convenient in-house 3D printing for sustainability and improving the mechanical and biological properties in bone regeneration at clinical sites. These materials can be developed as sustainable fiber to print customized scaffolds promptly and cost-effectively using 3D printing technology.

## Figures and Tables

**Table 1:** PCL/β-TCP filament weight ratios.

Symbol	β-TCP/Total Weight (wt%)	β-TCP Powder (g)	PCL (g)
PCL-00	0	0	100
PCL-10	10	10	90
PCL-20	20	30	120
PCL-30	30	45	105

**Table 2:** Element distribution of PCL/β-TCP fibers from EDS analysis. Carbon, C; oxygen, O; calcium, Ca; phosphorous, P.

	C	O	Ca	P
PCL-00	64.5% ± 1.8	35.5% ± 1.8	0%	0%
PCL-10	62.2% ± 0.7	34.1% ± 2.8	2.1% ± 1.4	3.4% ± 0.4
PCL-20	57.9% ± 2.2	32.2% ± 3.5	4.7% ± 2.0	5.2% ± 3.4
PCL-30	56.9% ± 3.1	30.2% ± 2.4	6.2% ± 2.0	6.7% ± 3.9

## Data Availability

Data is contained within the article.
